# NMR relaxation properties of the synthetic malaria pigment β-hematin

**DOI:** 10.1038/s41598-017-15238-3

**Published:** 2017-11-06

**Authors:** Yves Gossuin, Philippe Okusa Ndjolo, Quoc Lam Vuong, Pierre Duez

**Affiliations:** 1 Biomedical Physics Unit UMONS, 25 avenue Maistriau, Mons, 7000 Belgium; 2 Therapeutic Chemistry and Pharmacognosy UMONS, 25 avenue Maistriau, Mons, 7000 Belgium

## Abstract

200 million patients suffer from malaria, a parasitic disease caused by protozoans of the genus *Plasmodium*. Reliable diagnosis is crucial since it allows the early detection of the disease. The development of rapid, sensitive and low-cost diagnosis tools is an important research area. Different studies focused on the detection of hemozoin, a major by-product of hemoglobin detoxification by the parasite. Hemozoin and its synthetic analog, β-hematin, form paramagnetic crystals. A new detection method of malaria takes advantage of the paramagnetism of hemozoin through the effect that such magnetic crystals have on Nuclear Magnetic Resonance (NMR) relaxation of water protons. Indeed, magnetic microparticles cause a shortening of the relaxation times. In this work, the magnetic properties of two types of β-hematin are assessed at different temperatures and magnetic fields. The pure paramagnetism of β-hematin is confirmed. The NMR relaxation of β–hematin suspensions is also studied at different magnetic fields and for different echo-times. Our results help to identify the best conditions for β–hematin detection by NMR: *T*_2_ must be selected, at large magnetic fields and for long echo-times. However, the effect of β-hematin on relaxation does not seem large enough to achieve accurate detection of malaria without any preliminary sample preparation, as microcentrifugation.

## Introduction

In 2015, 200 million patients were suffering from the tropical disease malaria, and half a million died from the infection the same year^1^. The protozoan parasites of the genus *Plasmodium* causing the disease are transferred to humans by *Anopheles* mosquito bites. In the fight against malaria, reliable diagnosis tools are crucial since they allow the early detection of the disease, which increases the efficiency of the treatments, but also because they prevent overprescription of antimalarial drugs which may lead to drug resistance. The standard diagnosis method consists in the examination of Giemsa stained blood smears by light microscopy, which requires efficient microscopes and trained microscopists. Polymerase Chain Reaction (PCR) analysis of DNA extracted from the patient’s blood is also very sensitive, but rather expensive and difficult to use on the field. As the disease is mainly present in developing countries, the diagnosis tests must be rapid, cheap and easy to implement. Therefore, rapid diagnosis tests (RDTs) – based on lateral flow immunochromatographic detection of malaria antigens – were created and are now widely used^2,3^ even if their sensitivity is clearly lower than that of microscopy and PCR^4,5^. In this context, the development of new, rapid and sensitive diagnosis tools remains an important research area. Some groups focused on the detection of a major by-product of malaria infection, hemozoin. Indeed, during their proliferation in red cells, the parasites feed on haemoglobin, which results in the release of free heme. This redox agent being toxic, the parasite transforms it into an insoluble polymer, hemozoin, the so-called *“malaria pigment”*. Hemozoin and its synthetic analog, β-hematin, have comparable structural properties but their immunogenic properties may differ because of different crystal sizes and/or shapes^6–9^. They are both paramagnetic because they contain Fe^3+^ ions in high-spin configuration^10–13^. Values of the magnetic susceptibility of hemozoin and β-hematin were published by Brémard *et al*.^10^, Bohle *et al*.^11^, Hackett *et al*.^14^ and Butykai *et al*.^15^. In 2016, a study^16^ surprisingly proposed a superparamagnetic model for β-hematin, which however remains to be confirmed. The magnetic properties of hemozoin are at the origin of two detection methods which were recently proposed. The first method^15,17^ uses the magnetic moment of the pigment crystals to align them in a specific direction which makes them magnetically driven optical polarizers. The second method, evaluated by two different groups^18,19^, tries to take advantage of the paramagnetism of hemozoin through the effect that such magnetic crystals can have on Nuclear Magnetic Resonance (NMR) properties of neighbouring water protons in whole blood. Indeed, magnetic nano- and micro-particles cause a shortening of the relaxation times *T*_1_ and *T*_2_ of water protons, a phenomenon which is at the origin of the use of iron oxide particles as Magnetic Resonance Imaging (MRI) contrast agents. Karl *et al*.^19^ were the first to show the influence of hemozoin on water transverse relaxation time *T*_2_. Even if the effect was noticeable, they concluded that it was unlikely that this technique could achieve the requested sensitivity to become an efficient diagnosis tool on the field, since they were only able to detect parasitemia levels higher than 10,000 parasites per µl of blood, corresponding to 0.2% infected Red Blood Cells (RBC). A few years later, Peng *et al*.^18^ and Kong *et al*.^20^ reported a new technique for the rapid and sensitive detection of *Plasmodium* infected RBC using the measurement of *T*_2_. Their micromagnetic resonance relaxometry system uses the transverse relaxation of RBC pellets – obtained after microcentrifugation of a patient’s blood sample – to detect parasitemia levels of less than 10 parasites per µl, which is comparable to the detection limit of light microscopy, the gold standard. The marked discording conclusions of the two studies led to a reaction of Karl *et al*.^21^ who questioned the sensitivity reported by Peng *et al*.

In this work, the magnetic properties of two types of β-hematin will be assessed, to discriminate between paramagnetic and superparamagnetic behaviours, and the NMR relaxation of β-hematin-containing suspensions will be studied at different magnetic fields, in order to determine the best experimental conditions for the hemozoin and malaria detection by NMR.

## Results

### Electron Microscopy

**Figure 1:**
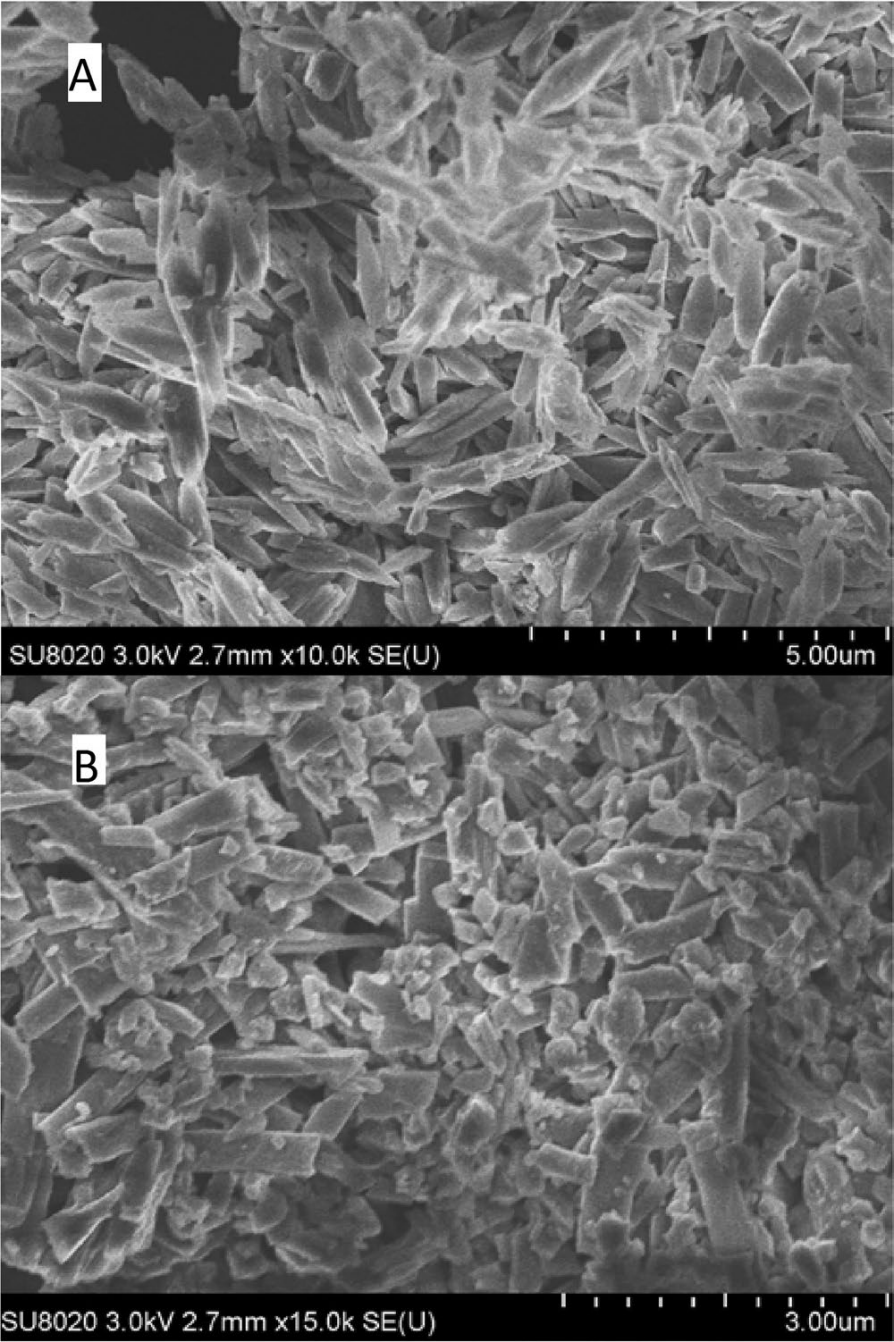
Scanning electron microscopy of (**A**) commercial β-hematin and (**B**) β-hematin synthesized in this work.

Figure 1 shows the electron microscopy images of commercial and home-made synthetic β-hematin (“Mons sample”). As expected^22^, the crystals present a rod shape. An estimation of the mean size and standard deviation of the crystals long axis was obtained by measuring 60 particles. For the commercial sample, L = 1.01 ± 0.34 µm while for the Mons sample L = 0.67 ± 0.28 µm. This is slightly over the size of *Plasmodium falciparum*, *P. vivax*, *P. ovale* and *P. malariae* hemozoin crystals (long axis, 0.30 to 0.50 µm)^23^.

### Magnetometry

**Figure 2:**
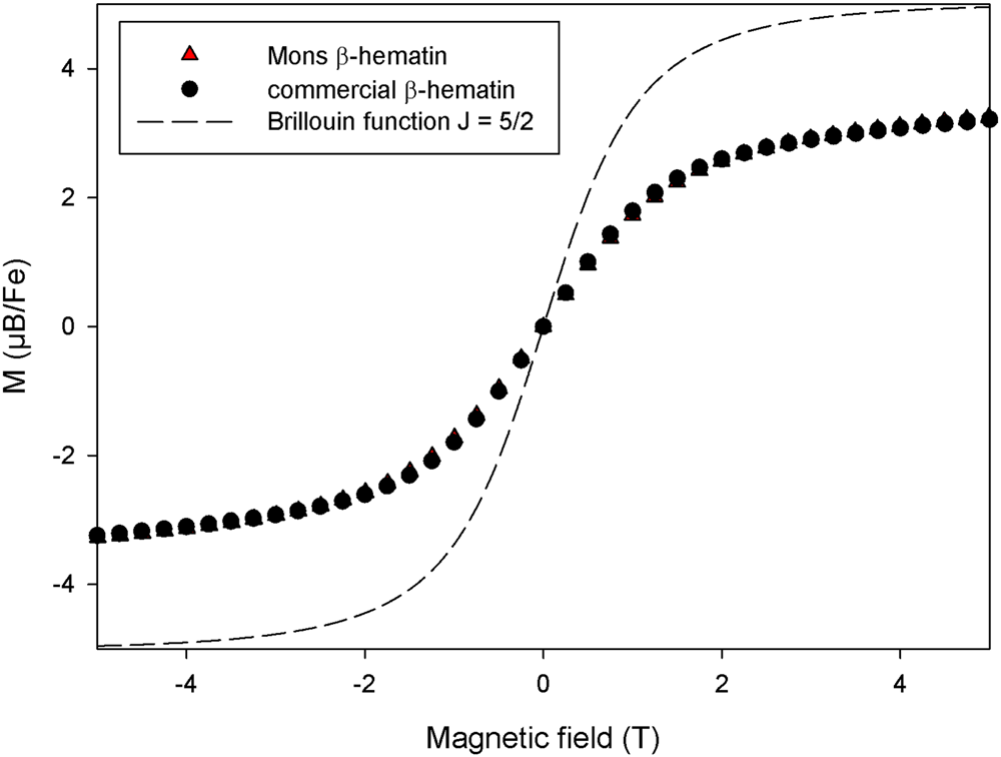
Evolution of the magnetization of the samples (expressed in Bohr magneton per iron ion) with the magnetic field at T = 1.85 K.

**Figure 3:**
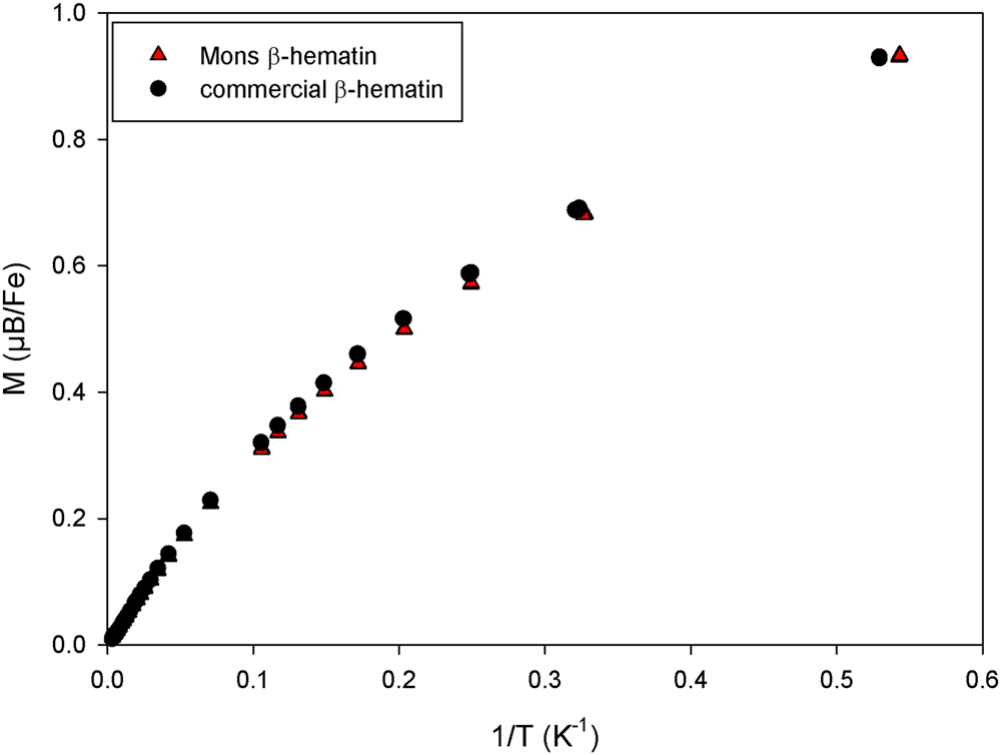
Evolution with 1/*T* of the magnetization at 0.5 T (expressed in Bohr magneton per iron ion).

Figure 2 shows the dependence of the β-hematin samples magnetisation (expressed in Bohr magneton per iron ion) on magnetic field strength at 1.85 K. The curves do not present any hysteresis and are similar for both samples. The shape of the curves does not correspond to a Brillouin function, because of the anisotropy of the iron magnetic moments in the β-hematin crystals^15^. Our data are in agreement with previously published data obtained at 2 K^15^. A good agreement is also obtained when looking at the evolution of the magnetisation at 0.5 T with the inverse of temperature (1/T) (Fig. 3). Finally, the room temperature susceptibility of our samples is clearly comparable to what was previously reported for β-hematin and hemozoin (Table 1). However, our results are clearly not in agreement with the data of Inyushin *et al*.^16^ who observed a Langevin dependence of the magnetization with the field at room temperature, with a saturation magnetisation of about 60,000 A/m while we obtain a non-saturated magnetization of 450 A/m in similar conditions at 1.5 T, more than two orders of magnitude less.

**Table 1:** Mass magnetic susceptibility of β-hematin and hemozoin at 298 K.

Sample	Mass susceptibility at 298 K (10^−7^ m^3^/kg)
Commercial β-hematin – this study	2.6
Mons β-hematin – this study	2.69
Hemozoin, Brémard *et al*.^10^	2.71
β-hematin, Hackett *et al*.^14^	3.05
Hemozoin, Hackett *et al*.^14^	2.58
β-hematin, Bohle *et al*.^11^	2.7

### NMR relaxometry

**Figure 4:**
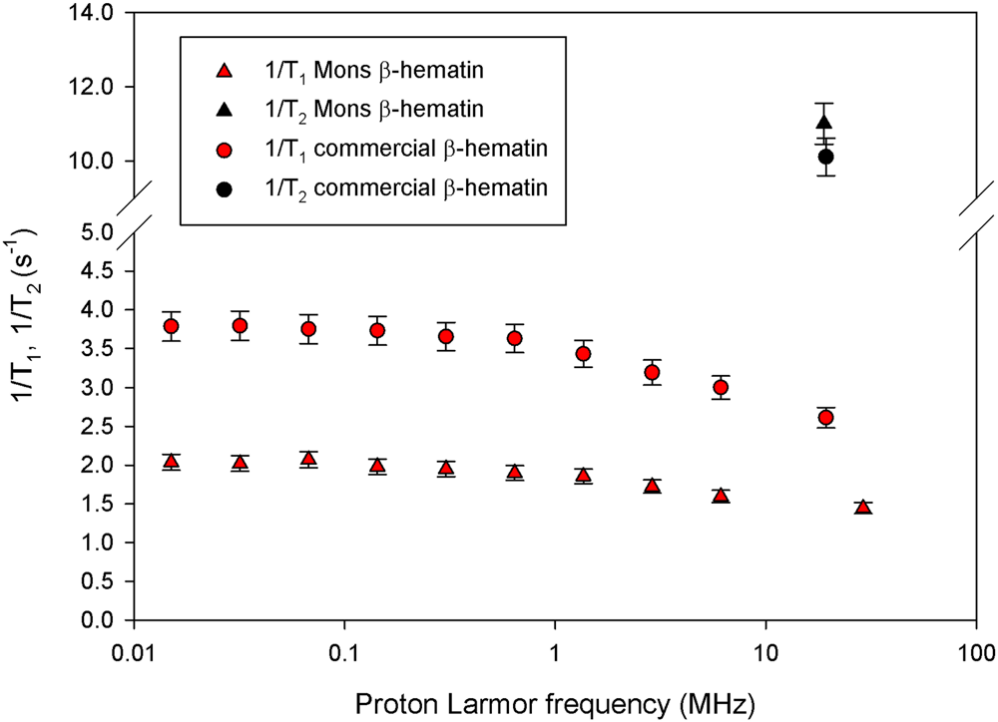
Longitudinal NMRD profiles of β-hematin suspensions (7.2 mg/ml) at T = 25 °C. Values of 1/*T*_2_ at 19 MHz are also given for comparison.

**Figure 5:**
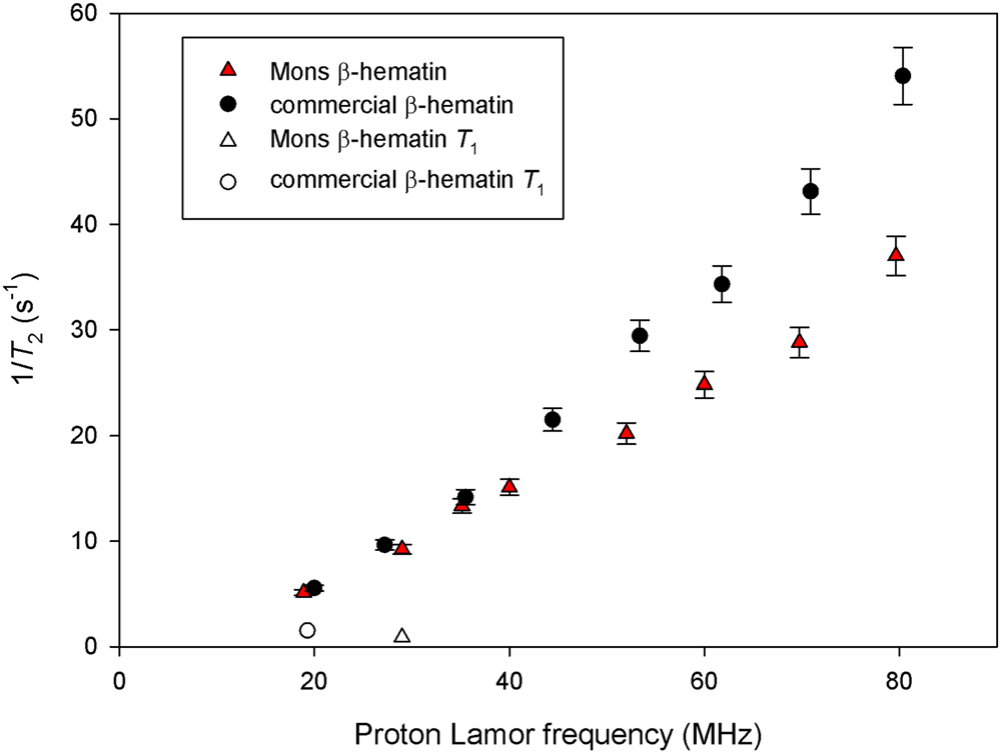
Evolution with the field of the transverse relaxation rate 1/*T*_2_ of β-hematin suspensions (3.88 mg/ml) at T = 25 °C. The interecho time is 1 ms.

**Figure 6:**
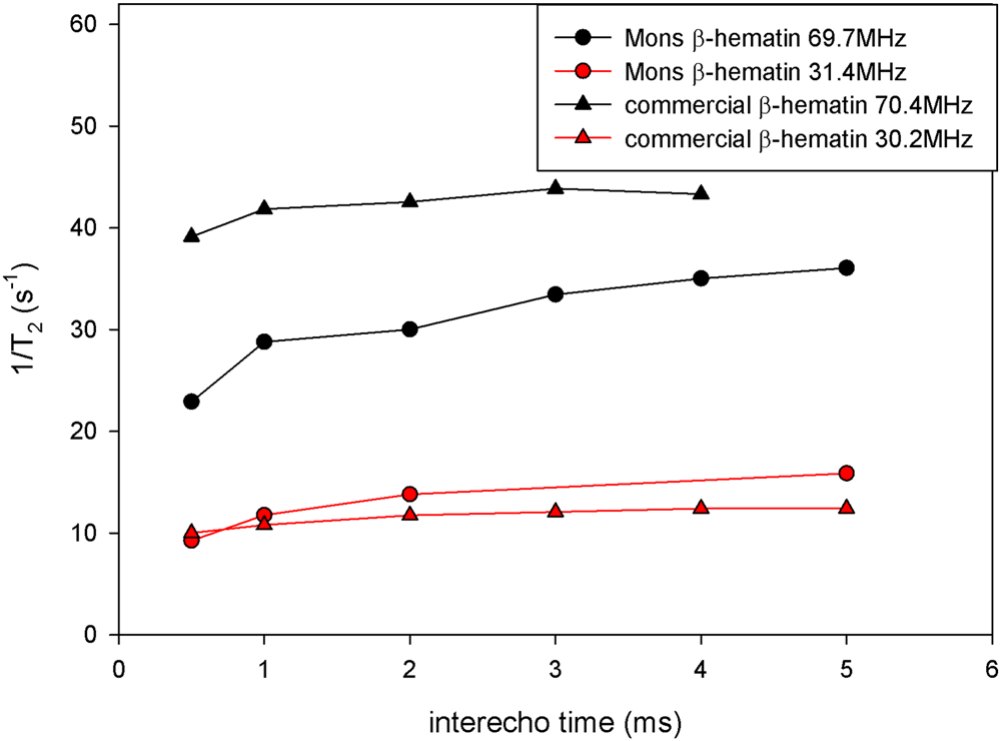
Influence of the interecho time on the transverse relaxation rate 1/*T*_2_ of β-hematin suspensions (3.88 mg/ml) at T = 25 °C and for two different magnetic fields.

Figure 4 presents the *T*_1_ NMRD profiles – the curve showing the evolution of the longitudinal relaxation rate with the magnetic field – of β-hematin suspensions. 1/*T*_1_ monotonously decreases with the field for both samples with a broad dispersion after 1 MHz, similar to what was observed for methemoglobin^24^. The effect of β-hematin on the longitudinal relaxation of water is really weak. Indeed, the relaxation rate (1/*T*_1_) normalised by the β-hematin concentration (in mg/mL) is always smaller than 0.55 s^−1^ mL mg^−1^. When expressed in terms of relaxivity – relaxation rate normalized by the iron concentration in mM – it gives less than 0.35 s^−1^ mM^−1^ which is far below the relaxivities of usual MRI contrast agents. This is also an order of magnitude smaller than the relaxivity of methemoglobin^25^. The transverse relaxation of water protons is more affected by the presence of β-hematin crystals as shown in Fig. 5. For both samples, the transverse relaxation rate 1/*T*_2_ significantly increases with the field. This increase is more important for the commercial β-hematin compared to the Mons β-hematin. Such an increase reflects the field-dependent increase of the magnetic moment of the crystals. Their effect on transverse relaxation becomes stronger when they present larger magnetic moments. This is consistent with the predictions of the classical relaxation theories^26^. The transverse relaxation rate normalised by the β-hematin concentration (in mg/mL) are given in Table 2 for our samples at 20 MHz and 60 MHz together with values obtained from the literature. Finally, the influence of the interecho time on the transverse relaxation was also investigated. Transverse relaxation is more efficient for large interecho times as it is often the case for magnetic compounds (Fig. 6). The effect is more pronounced for the Mons sample. The differences of NMR results between the two samples can easily be explained by the differences of size and shape of the β-hematin crystals in the samples. It should here be stressed that the effect of paramagnetic crystals on the NMR relaxation of water protons is dependent on many parameters, not only on the magnetic susceptibility of the particles.

**Table 2:** Normalized transverse relaxation rates of β-hematin samples at 25 °C for two different magnetic fields (∼20 and 60 MHz).

Sample	Normalised 1/*T*_2_ at 20 MHz (s^−1^ mL mg^−1^)	Normalised 1/*T*_2_ at 60 MHz (s^−1^ mL mg^−1^)
Commercial β-hematin – this study	1.43 ± 0.05	8.84 ± 0.27 *(at 62 MHz)*
Mons β-hematin – this study	1.32 ± 0.05 *(at 19 MHz)*	6.39 ± 0.19
β-hematin, Karl *et al*.^19^	—	8.3
Hemozoin, Karl *et al*.^19^	—	7.7
Hemozoin after centrifugation, Peng *et al*.^18^	230	—

## Discussion

Our results confirm the pure paramagnetism of β-hematin. It seems the superparamagnetism reported recently^16^ had another origin than the presence of β-hematin; this may come from a contamination of β-hematin by a superparamagnetic agent, such as hematite^27^. This clearly impacts the potential of conventional NMR relaxometry for the detection of malaria: paramagnetic particles are much harder to detect than superparamagnetic ones.

From the NMR point of view, graph 4 shows that transverse relaxation has to be used, since longitudinal relaxation is far too small. At 60 MHz, the normalized transverse relaxation rate obtained in this work (Table 2) is in good agreement with the value reported by Karl *et al*.^19^ for β-hematin suspensions. But our data bring more information: 1/*T*_2_ increases with the field (Fig. 5) and also with the interecho time used in the CPMG sequence (Fig. 6). Using a large magnetic field is better, but it is hardly feasible in small and low-cost NMR systems. For example, the micromagnetic resonance system proposed by Peng used a field of 20 MHz. Moreover, long echo times are preferable to yield large values of 1/*T*_2_, but they cannot be used in those micro-NMR systems whose magnetic field homogeneity is rather bad. For such systems, the purely instrumental dependence of *T*_2_ on the echo time would mask the effect of β-hematin. This is why Peng *et al*. used an interecho time of 60 µs, a value smaller than the lowest value we tested.

A clear limitation of our study is the use of simple aqueous suspensions of β-hematin crystals instead of whole blood samples. The transposition of our conclusions to blood may not be straightforward. But to understand what is happening in blood, we believe that a first and necessary step is the study of simpler system, i.e. aqueous suspensions. In order to compare our results with those obtained for infected RBC samples containing hemozoin (from Karl *et al*. and Peng *et al*.), the following approximation was applied. Assuming that, in each parasitized RBC, 50% of hemoglobin has been converted into hemozoin, one can roughly estimate that an hemozoin content of 30 µg hemozoin/ml corresponds to a 1% parasitemia, as shown in Newman *et al*.^17^. This allows to normalize the 1/*T*_2_ values obtained for infected RBCs samples (Table 2). The results of Karl *et al*. for hemozoin are in good agreement with our results for β-hematin. Considering only those results, it seems that conventional NMR relaxometry *alone* cannot be used for a sensitive diagnosis of malaria through detection of *Plasmodium* species: the normalized transverse relaxation rates of hematin/hemozoin are too small. This fully agrees with the conclusions of Karl *et al*.^19^ but not with those of Peng *et al*.^18^ who achieved an excellent sensitivity. Indeed, from Fig. 2a of the latter study, one can estimate that, for a parasitemia level of 1%, the transverse relaxation rate increase measured after centrifugation was 7 s^−1^. This leads to a normalized transverse relaxation rate which is about 170 times larger than what was obtained in this work for the same magnetic field. This difference in the normalized relaxation rate clearly explains the diverging conclusions of Karl *et al*. and Peng *et al*. The large value of the normalized relaxation rates obtained by Peng *et al*. may be related to the microcentrifugation step they applied before the NMR measurements. Indeed, centrifugation concentrates the infected red blood cells that present a higher density than the normal red blood cells. Moreover, in real blood, maybe the use of ultra-short echoes allows to probe other compartments of protons - like macromolecular protons^28^ - which could be more affected by the presence of hemozoin? Anyway, without preliminary centrifugation of the sample and the use of ultra-short echoes, the sensitivity of the NMR method would have been much worse. This is clearly something to take into account when trying to develop/adapt NMR methods for the detection of malaria: conventional relaxometry alone is not enough.

## Methods

### Samples

A sample of 5 mg of β-hematin (lot HMZ-38-01) was purchased from InvivoGen (Toulouse, France). This sample is referred to as “commercial β-hematin” throughout the paper.

A second sample of β-hematin was synthesized according to published protocols^29,30^ with slight modifications; briefly, a solution of 4.54 mM porcine hemin (Sigma-Aldrich, >98% pure) in 0.04 M NaOH was adjusted to pH 4.0 with 2% propionic acid dropwise and incubated at 70°C for 18 h. The formed crystals were filtered on cellulose, washed with 1 M acetic acid, dried over phosphorus pentoxide, manually powdered and stored at 4°C. The powder was characterized by infrared spectroscopy, yielding 3 bands characteristic of hemozoin^7^ at 1711, 1662 and 1209 cm^−1^. This sample is referred to as “Mons β-hematin” throughout the paper.

### Electron microscopy

Electron microscopy images were acquired directly on the powders with a Hitachi Scanning Electron Microscope, model SU8020 using a 3 kV voltage.

### Magnetometry

All the magnetic measurements were carried out on a mini high field measurement system from Cryogenics Limited (London, UK) with the vibrating sample option. The maximum field is 5 T and the minimum temperature is 1.7 K. The measurements were directly performed on powders of β-hematin. When the diamagnetic contribution of the sample holder was non negligible (more than 1% of the total magnetic moment), it was subtracted from the raw data. It was especially the case at low fields and high temperature. For the comparison of the susceptibility values with the literature data, the density of β-hematin was needed. We used the value of 1440 kg/m^3^ reported by Coronado *et al*.^8^. The molecular weight of β-hematin was taken as 633.5 g/mol.

### NMR relaxometry

For NMR results, the magnetic field is expressed in terms of the proton Larmor frequency: a field of 1 Tesla corresponds to a Larmor frequency of 42.6 MHz. The suspensions were sonicated during 3 min before the session of NMR measurements and the tubes were vigorously shaken by hand before each NMR measurement. Low-field NMRD profiles (*T*_1_) of aqueous suspensions were measured from 0.015 to 40 MHz with a Spinmaster fast field-cycling relaxometer (STELAR, Mede, Italy) using 600 µL of suspensions in a dedicated NMR tube. The *T*_2_ and *T*_1_ measurements at higher fields were carried out on a homemade variable field relaxometry system using an electromagnet and a lapNMR RF spectrometer covering a range of Larmor frequency from 10 to 90 MHz. The CPMG sequence was used, with an interecho time of 1 ms unless other mention. The temperature of all the NMR measurements was 25 °C ± 1°C.

### Data Availability

All data generated or analysed during this study are included in this published article.
